# Empowering Melatonin Therapeutics with *Drosophila* Models

**DOI:** 10.3390/diseases9040067

**Published:** 2021-09-26

**Authors:** Cassandra Millet-Boureima, Caroline C. Ennis, Jurnee Jamison, Shana McSweeney, Anna Park, Chiara Gamberi

**Affiliations:** 1 Biology Department, Concordia University, Montreal, QC H4B 1R6, Canada; cassandra.millet@mail.concordia.ca; 2 Department of Biology, Coastal Carolina University, Conway, SC 29528-6054, USA; ccennis@clemson.edu (C.C.E.); jbjamison@coastal.edu (J.J.); skmcsween@coastal.edu (S.M.); apark309@gatech.edu (A.P.)

**Keywords:** melatonin, oxidative stress, *Drosophila*, longevity, PKD, neurodegeneration

## Abstract

Melatonin functions as a central regulator of cell and organismal function as well as a neurohormone involved in several processes, e.g., the regulation of the circadian rhythm, sleep, aging, oxidative response, and more. As such, it holds immense pharmacological potential. Receptor-mediated melatonin function mainly occurs through MT1 and MT2, conserved amongst mammals. Other melatonin-binding proteins exist. Non-receptor-mediated activities involve regulating the mitochondrial function and antioxidant cascade, which are frequently affected by normal aging as well as disease. Several pathologies display diseased or dysfunctional mitochondria, suggesting melatonin may be used therapeutically. *Drosophila* models have extensively been employed to study disease pathogenesis and discover new drugs. Here, we review the multiple functions of melatonin through the lens of functional conservation and model organism research to empower potential melatonin therapeutics to treat neurodegenerative and renal diseases.

## 1. Introduction

Melatonin (5-methoxy N-acetyltryptamine), nicknamed the “hormone of darkness”, was first discovered in the cow pineal gland, and has, since, been found in several mammals and other organisms, including plants (reviewed in [1]). Compared to mammalians, much less is known about how melatonin functions in invertebrates and plants [2]. Responsible for maintaining a steady circadian rhythm, melatonin is primarily synthesized and secreted into the blood by the mammalian pineal gland of the brain, peaking at night and reaching its lowest during the day [2,3,4,5,6,7,8,9,10,11,12]. Melatonin is also secreted by the retina, the lacrimal and Harderian glands, gut, bone marrow, platelets, and thyroid [13,14,15,16,17]. Finally, several (and potentially all) cells produce melatonin in the mitochondria [18,19], and, likely, the cytoplasm [20] with a distinct, non-circadian rhythmicity. Melatonin has been found to influence many cellular pathways and organismal functions [14,17]. Its effects are prominent on the circadian cycle, as well as endocrine function, immunity, and fertility [21]. Moreover, melatonin protects from arterial vasoconstriction and neurodegeneration via at least two types of receptors found in many cell types [22]. Melatonin effectively scavenges reactive oxygen species (ROS) and functions as a mitochondrial and cyto-protector [23,24]. The observed reduction in melatonin levels in several diseases, including neurodegeneration and cancer, constitutes the foundation for pursuing melatonin as a therapeutic. Melatonin is amphipathic and can easily cross cell membranes [25]. Therefore, it is expected to work through binding to transmembrane receptors, intracellular proteins, possibly to nuclear receptors, as well as display antioxidant activity [26].

Melatonin appears to have originated from photosynthetic bacteria, primitive cyanobacteria and alpha-proteobacteria before the endosymbiotic events believed to have originated eukaryotic mitochondria and chloroplasts [27]. Melatonin can donate electrons easily [28,29] and may have initially functioned as a free radical scavenger to reduce oxidative stress from photosynthesis and metabolism [2,27,30,31]. Organismal evolutionary diversification prompted an increased range of melatonin functions beyond its fundamental antioxidant capacity, to include the regulation of circadian rhythm, sleep, ciliary swimming behavior, vision, immunity, and more [2,32].

In contrast to the long evolutionary history of melatonin, its transmembrane receptors appeared relatively late in evolution [2]. Specifically in mammals, melatonin receptor 1 (MT1) and melatonin receptor 2 (MT2) display high affinity for melatonin and belong to the G-protein-coupled receptors (GPCR) superfamily [2,33,34,35,36,37,38]. A third receptor, MT3, identified as a binding site on the cytosolic detoxification enzyme quinone reductase 2 (QR2), is also found in various tissues, including liver, heart, lungs, kidney, intestine, and muscle [26,39,40,41].

Highly conserved functions and ubiquitous effects within the body make melatonin a primary research interest with potential pharmacological application. However, its precise molecular mechanism(s) of action, its reception and signal transduction have yet to be fully elucidated. Model organisms such as the fruit fly *Drosophila melanogaster* can recapitulate fundamental biological mechanisms, provide mechanistic insight, and contribute to the evolving understanding of melatonin function and its possible use as a therapeutic.

## 2. Melatonin Receptors

Encoded by melatonin receptor genes found, respectively, on human chromosome 4 and 11, the MT1 (alias MTNR1A, Mel1a) and MT2 (alias MTNR1B, Mel1b) proteins bind melatonin and share a high degree of sequence homology [37,42]. MT1 and MT2, respectively consisting of 350 and 362 amino acids (aa), share a 55% overall aa homology and 70% homology within the transmembrane domains [32]. Both have seven transmembrane α-helices connected by alternating loops with the amino-terminus on the extracellular side and the carboxy-terminus on the intracellular side [41]. The recently reported crystal structures of human MT1 and MT2, unexpectedly revealed the presence of highly specific orthosteric binding sites for melatonin buried within the membrane that, due to melatonin amphipathic properties, may contribute to ligand specificity [43].

While both MT1 and MT2 are high affinity melatonin receptors, the human MT2 exhibits a lower affinity for ^125^I-melatonin than MT1 [34]. Human MT1 and MT2 can form homo- and heterodimers with each other and heteromers with other GPCRs, consistent with them displaying several cellular functions (reviewed in [44]). MT1 and MT2 heterodimers are common even in the absence of a ligand and conformational changes have been shown to occur upon ligand binding [45]. Oligo- and heteromerization greatly impact the functional diversity of these receptors [46]. MT1 and MT2 primarily remain coupled to Gi/o proteins and display a high affinity for melatonin due to the formation of the ligand–receptor–G-protein (L–R–G) (or β-arrestin) complex [46]. Several other signaling molecules and G proteins have been reported to interact with MT1 and MT2 in specific cases [46]. Dissimilar to MT1 and MT2, the MT3 receptor exhibits a low binding affinity for melatonin [47,48]; however, its inhibition is theorized to play a role in the antioxidant effects of melatonin [49]. An analysis of *MTNR* genes encoding the melatonin receptors from 45 vertebrate species provided insight into their origin and evolution. In particular, the *mtnr1c* gene found in lower mammals contains a C-terminal expansion, and is thought to have evolved after the evolutionary branching from Monotremata and Marsupialia [50]. Interestingly, the orphan receptor GPR50 appears to be a mammalian *mtnr1c* ortholog. Considering the two rounds of whole genome duplications occurred since the common vertebrate ancestor [51], the loss of the ancestral *mtnr1c* function, and the analysis of the regions adjacent to the *MTNR* genes, it seems likely that during the recent tetrapod evolution several melatonin receptor-related genes may have been lost [50]. Numerous sequences in GenBank have been automatically annotated as encoding “melatonin-receptor-like” proteins, including entries from invertebrates and insect species. However, their divergence and current lack of functional evidence cautions against considering them as bona fide melatonin receptors. Despite structural similarities in the MT1, MT2, and GPR50 transmembrane motifs, GPR50 in humans does not bind melatonin directly, but inhibits MT1 receptor functions via heteromerization [46,52]. Transcription factors belonging to the retinoic acid-related orphan nuclear hormone receptor (ROR) family are functionally linked to melatonin pathways; however, their capacity to bind melatonin directly is debated [53].

### 2.1. Melatonin Receptor Functions

Sleep/wake homeostasis and circadian rhythm regulate sleep (reviewed in [54]). In mammalians, the master circadian clock is located within the suprachiasmatic nucleus (SCN) in the hypothalamus, where melatonin is released and controls SCN activity through the MT1 and MT2 receptors [55,56]. Melatonin receptors seem to be involved in a plethora of physiological activities and cellular processes (reviewed in [22,34]) as diverse as phototransduction and ocular growth [57,58], blood pressure regulation [59], immunomodulation [60,61], the hair cycle [62], and may exhibit oncostatic effects in several cancers [34]. Additionally, MTNR1A was found to have regulatory effects on reproductive seasonality that appears tightly linked to the light/dark cycle [63]. Tissue-specific expression of receptors relates to several melatonin physiological effects, yet it has only been partly defined. MT1 and MT2 are expressed in the cell membranes of a variety of tissues throughout the body, including the brain, retina, cardiovascular system, liver, gallbladder, colon, skin, kidneys, among others [64], (reviewed in [22]). To date, the mammalian melatonin receptors have been studied extensively. Some well-described melatonin receptor functions are listed in Table 1. In contrast, melatonin signaling and receptor homologs in other species, including invertebrates, remain largely unknown.

In the central nervous system (CNS), MT1 and/or its mRNA are found in the SCN, pars tuberalis (PT) [89,90,91], and the retina [57]. MT2 is expressed in the olfactory bulb, forebrain, hippocampus, amygdala, and superior colliculus [92], as well as in the septum, within specialized cells of the hippocampus, the pars reticulata of the substantia nigra, ventral tegmental nucleus and other regions associated with non-rapid eye movement (NREM) sleep [90]. *MT2* mRNA is found in the retina, sclera, lens, and retinal pigment epithelial cells [93]. Although both MT1 and MT2 receptors were detected in the retina and are essential for retinal function, the *MT2* mRNA was, surprisingly, not detected within the photosensitive retinal ganglion cells in mice [93]. Of note, mice are nocturnal. Melatonin synthesis is similar in both nocturnal and diurnal animals, with peak release being recorded during the middle of the night (12–3 am) [94]. It has been shown that melatonin induces typical nighttime behavior, i.e., activity for nocturnal animals, and sleep for diurnal species such as humans [95,96,97] (reviewed in [98]).

The simultaneous activation of MT1 and MT2 can elicit diverse responses with additive, cooperative or opposing effects [38]. In vertebrates, the light/dark cycle affects the retina, which signals to the master circadian clock in the SCN via the retino-hypothalamic tract [99]. Responding to the SCN, the pineal gland synthesizes and releases melatonin that is captured by the target cells through MT1 and MT2 receptors (reviewed in [100]). Creating a regulatory feedback loop, the SCN cells, which express MT1 and MT2, also respond to melatonin (reviewed in [56]). MT1 and MT2 are cyclically expressed daily during the light/dark cycle [101]. Melatonin promotes the expression of clock genes, which is expected to contribute to its circadian functions [102] and may also operate in receptor-independent ways [103], or through interaction with other receptors [87,104,105]. Several GPCRs become desensitized upon ligand binding, which helps to regulate the intensity of cell response. While there is suggestive evidence of such properties for MT1 and MT2 (e.g., [106,107]), both mechanism and physiological roles remain unknown. Genetic studies with murine knockout animals have indicated that MT1 and MT2 may regulate REM sleep and NREM sleep phases, respectively [87]. The relationship between melatonin, MT1, MT2, and sleep appears complex. The genetic and pharmacologic manipulation of the MT1 and MT2 pathways through single and double MT1/MT2 knockout and chemical probing employing agonists, as well as the study of the effects of the surgical removal of the pineal gland, collectively suggest that melatonin contributes to the sleep wake cycle, yet, may not be obligatory [87,105]. The double MT1/MT2 knockout mice displayed an almost normal sleep phase duration but may have altered cycling between NREM and REM sleep [87]. Because both receptors are differentially expressed in the brain areas controlling REM and NREM sleep, one intriguing possibility is that MT1- and MT2-dependent responses to melatonin may be linked. This, together with potentially differential receptor deactivation cycles during melatonin peak time, may yield the REM/NREM cycling [55,87,108]. The mechanism remains, however, to be fully demonstrated experimentally.

In the cardiovascular system, melatonin regulates smooth muscle and endothelial cells [75,109]. Melatonin receptors may increase coronary blood flow and improve cardiac function [109]. Depending on the tissue, melatonin may also induce vasoconstriction or vasodilation. In smooth muscle and coronary arteries, MT1 appeared to mediate vasoconstriction while MT2 activation induced vasodilation [75,77,110], by reducing cyclic adenosine monophosphate (cAMP) levels and phosphatidylinositol 4,5-bisphosphate hydrolysis [111,112]. In animal studies, specific arteries responded differentially to melatonin exposure. Rat and pig coronaries constricted [113], while the rabbit pulmonary, aorta, iliac, and renal arteries dilated [75,77,110,114,115,116,117,118,119]. In humans, while renal blood flow and conductance decreased in response to melatonin, forearm vascular beds exhibited the opposite response, and cerebral circulation was unaffected [120]. Pharmacological studies suggest that some of these effects may be independent of MT1 and MT2 receptors [121]. Studies in pigs also imply that in endothelial cells, MT2 activation may increase nitric oxide (NO) production, which in turn causes vasodilation [109]. However, melatonin antioxidant properties are largely responsible for suppressing NO production in addition to significantly increasing superoxide dismutase (SOD) activity [122,123,124]. While this prompts melatonin’s potential use in treating diseases such as Alzheimer’s disease, further studies will be needed to decipher its effects on endothelial cells.

Melatonin may also impact immunity [60,61]. High levels of melatonin have been shown to promote immune system functions, while, conversely, low levels are associated with the suppression of numerous immune parameters. The discovery of melatonin receptors in multiple lymphoid organs and lymphocytes indicates that there may be multiple mechanisms of action [21]. Mice lacking a functional *MT1* gene were used to show that MT2 receptors are responsible for the melatonin enhancement of splenocyte proliferation and the regulation of anti-keyhole limpet hemocyanin (KLH) IgG concentrations [81,125]. Note, that the T-cell-dependent KLH antigen from mollusks is widely employed in immunotoxicology to evaluate immune function in varying conditions (reviewed in [126]). Additionally, the activation of MT2 receptors has been shown to inhibit melanoma cell growth [127].

Due to its high cell permeability, free melatonin in humans can bind to intracellular MT3 and nuclear ROR proteins [128]. MT3 (alias ML2, NQO2) is a quinone reductase expressed in several vital organs, including kidney, liver, heart, and lung and in muscle, intestine, and brown fat, that inhibits the quinones electron transport chain and protects from oxidative stress (reviewed in [34,40]).

Melatonin has one possible nuclear receptor type in the ROR protein family. The ROR/RZR proteins are zinc-finger transcription factors with α, β, and γ subgroups. Dissimilar to RORβ, both RORα and RORγ are known to participate in several pathways that are also regulated by melatonin. For example, the transcriptional regulation of the clock gene *Bmal1* by RORα correlates with the mammalian circadian rhythm and has been shown to be necessary for normal circadian regulation in mice [129,130]. Melatonin promotes RORα transcriptional activity and can be co-immunoprecipitated with RORα [131], indicating some functional interaction. Physiological studies also point to a large functional overlap between the two; however, their direct binding is controversial (reviewed in [53]). Based on some experimental observations, it has been proposed that RORα-mediated effects may be indirect and exerted through other factors, e.g., MT1, MT2, sirtuins, the redox state, mitochondria, and, possibly, the expression of ROR antagonist REV-ERB [132,133,134,135,136,137,138,139].

The orthologous GPR50 (alias H9, ML1X; found in vertebrates, except birds and fish) and Mel1c (in fish, amphibians, and birds) proteins are also GPCRs. GPR50 exhibits about a 50% sequence identity with MT1 and MT2; however, it does not appear to bind melatonin [44]. Interestingly, GPR50 can form heteromers with both MT1 and MT2 and negatively regulate the melatonin–MT1 interaction, while leaving MT2 binding activity intact [52] (reviewed in [40]).

### 2.2. Molecular Mechanisms of Melatonin-Receptor Signaling

MT1 and MT2 are activated by distinct physiological concentrations of melatonin released from the pineal gland due to feedback onto the SCN [41]. The pathways downstream of MT1 and MT2 affect intracellular cAMP and cGMP, calcium (Ca^2+^) levels, and the activation of specific protein kinases [34].

**Figure 1:**
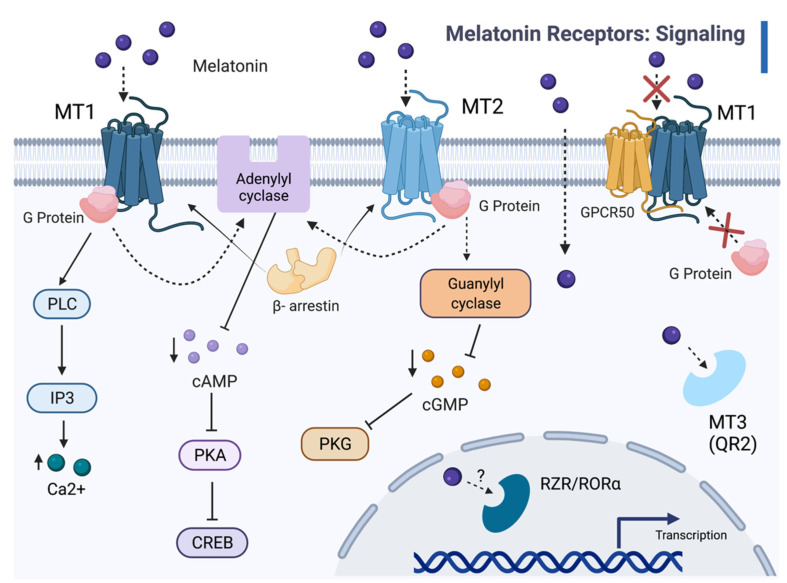
The melatonin signaling cascades. Melatonin binds to transmembrane receptors MT1 and MT2, as well as the MT3 binding site on the cytosolic enzyme QR2, and, possibly, the nuclear receptor RZR/RORα. Melatonin (ligand) binding with MT1 and MT2 receptors recruits β-arrestin and activates G proteins, which inhibit adenylyl cyclase activity and decrease cAMP levels. MT1 coupling to G proteins activates phospholipase C (PLC), which leads to increased intracellular Ca^2+^. Melatonin-dependent activation of MT2 and associated G proteins prompts interaction with guanylyl cyclase, which reduces cGMP levels; therefore, lowering PKG activity. In vertebrates, except for birds and fish [42,141], heteromerization of GPR50 and MT1 (but not MT2) inhibits both G protein interactions and melatonin binding. Melatonin acts as a ligand for MT3 and, possibly, for RORα, independently of the MT1 and MT2 pathways. With RORα and RORγ, melatonin affects nuclear transcription factor activity and gene expression.

Most notable among MT1 functions, is the inhibition of cAMP accumulation by the pertussis toxin (PTX)-sensitive G proteins in the mammalian pituitary and SCN [26,41,48,86,87]. MT1 engagement by melatonin activates Gi proteins, inhibits adenylyl cyclase activity, and decreases intracellular cAMP (Figure 1) [38,46]. Such a decrease activates protein kinase A and, subsequently, the transcription factor cAMP-responsive element binding (CREB) [38,46]. Additionally, the MT1 receptor augments potassium conductance through Inner Rectifier Potassium (Kir) channels, induces the mitogen-activated protein kinase 1/2 (MAPK), and extracellular signal-regulated kinase (ERK) 1 and 2 [38]. Circadian signaling regulates melatonin binding and the expression of *MT1* mRNA [34]. Both responses and the signal transduction pathways themselves variably correlated to the circadian rhythm. Studies with recombinant receptors illustrated the ability of MT1 to activate many types of G proteins [34]. MT2 is closely associated with the inhibition of adenylyl cyclase and guanylyl cyclase, as well as phosphoinositide production [48,88]. MT2 also inhibits forskolin-stimulated cAMP production in addition to cGMP formation and activates PKC in the SCN [38]. In (nocturnal) rodent SCN and PT, varying levels of *MT1* mRNA expression and ^125^I-melatonin binding are exhibited throughout the day, with greater expression during the daytime [140].

## 3. Receptor-Independent Melatonin Functions

In addition to its several receptor-mediated functions, melatonin can also function independently. A well-known example of a receptor-independent action is its activity to counter oxidative stress. Melatonin is an effective scavenger for free radicals formed in response to stressors activating the AP1 transcriptional response [142,143,144]. In mitochondria, melatonin functions to neutralize dangerous pro-oxidant metabolic byproducts of oxidative phosphorylation and the electron transport chain, such as radicals and peroxides [145,146]. Reportedly, the mitochondrial melatonin concentration (~100 nM) is approximately one hundred times that of the circulatory melatonin released by the pineal gland (1 nM at peak release) [147]. Such a concentration is achieved from a combination of rapid uptake and transport via the PEPT1/2 transporters [148] and glucose transporter 1 (GLUT1) [149], and endogenous synthesis independent of the circadian pineal release [18]. It has been proposed that mitochondrial melatonin synthesis reflects the capacity of the ancestral bacterial endosymbiont that modern mitochondria originate from (reviewed in [150]). In addition to its ROS scavenging power, melatonin affects several mitochondrial pathways. It improves the function of the electron transport chain [147] and ATP production [151]. It also promotes the synthesis of antioxidant glutathione [152], upregulates mitochondrial sirtuin SIRT3 [153,154,155], which, in turn, increases SOD antioxidant activity and acetyl coenzyme A synthesis. The latter is a necessary cofactor for a limiting melatonin biosynthetic enzyme, arylalkylamine N-acetyl transferase [156]. Altogether, these pathways appeared to reduce mitochondrial ROS damage in rat and mice models [157,158,159] (reviewed in [160]).

Experimental observations suggest that mitochondrial melatonin also exits this organelle [19] and may affect other cell districts and compartments, as well as nearby cells in paracrine fashion [150]. Because the mitochondrial membrane contains abundant MT1 and MT2 [19,161,162], mitochondria can respond to released mitochondrial melatonin in a way reminiscent of cellular autocrine loops.

**Figure 2:**
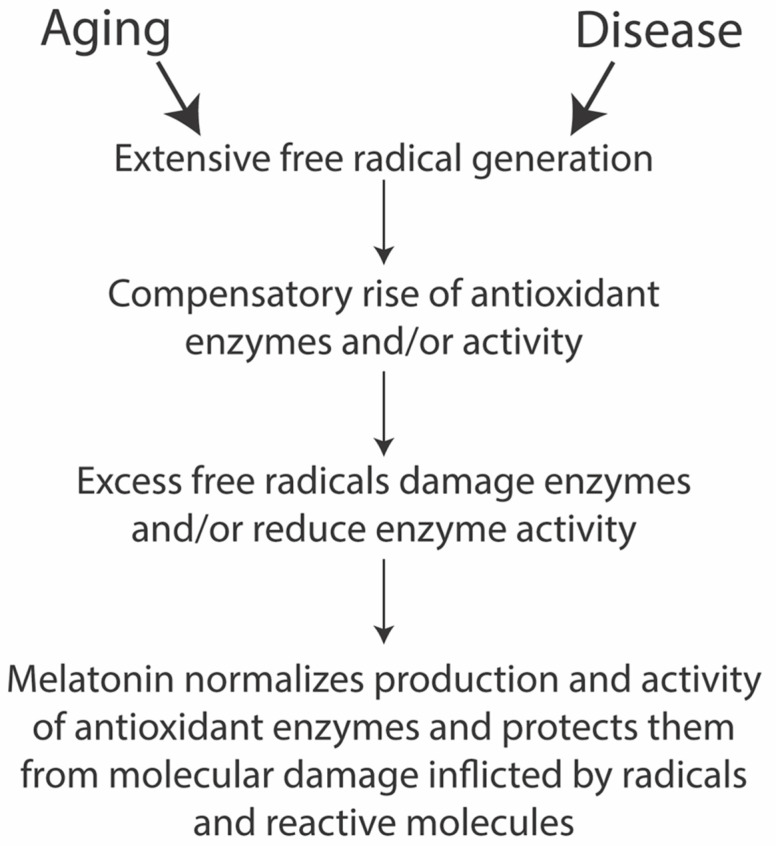
Simplified melatonin antioxidant cascade (drawn after [163]).

Several diseases and aging feature cellular oxidative damage (Figure 2). Cells appear to have a natural defense against these injuries in their endogenous melatonin production and melatonin signaling. However, the natural buffering capacity may become insufficient and, in such cases, melatonin administration in therapeutical dosages promises to help remedy such degeneration. Additionally, several diseases affect the mitochondria directly or compromise them indirectly (discussed below), in which case, melatonin may become an attractive treatment option.

### 3.1. Melatonin and Longevity

One of the risk factors for several prevalent diseases, biological aging is the decline in the physiological ability to perform or meet certain demands that occur as time passes [164]. Shared by most organisms and characterized by the accumulation of damage in processes at the molecular, cellular, and, eventually, the organ levels, biological aging increases individual susceptibility to disease, infection, and death. Aging is influenced by genetic, dietary, and environmental factors. Considering the social costs and degraded life quality associated with disease, preventing age-related conditions and prolonging a healthy life span are biomedical topics of great interest.

The genes found to affect survival displayed weak effects on longevity when assayed individually [165]. Consistently, genetic manipulation of the aging process in mice could only increase lifespan modestly [165]. Thus, aging appears to be only partly modulated by genetic factors. A series of observations support such conclusion. Aging may present differently even among individuals of the same species. In fact, in populations of genetically identical yeast or laboratory animals, individual age at death varies. Similarly, monozygotic human twins usually die at different ages [166]. Twin studies estimated that the individual genetic makeup may affect lifespan at most by 30% [167]. Aging is associated with many features, including genomic instability, telomere attrition, and mitochondrial dysfunction. During aging, melatonin production gradually declines [168] (reviewed in [169]). Life-extension protocols and environmental factors such as nutrition appeared to impact longevity at about 70%, more than twice the estimated impact of genetic makeup [170]. Thus, many compounds known to affect aging mechanisms have been tested for life-extending potential. The DrugAge database of aging-related drugs reports that pineal gland extract was found to be the most effective, prolonging mouse lifespan by about 31% [171,172]. Similarly, epithalamin, a tetrapeptide found in pineal gland extract, was also reported to elicit a nearly identical longevity extension [171,173]. Epithalamin was found to be active in both its natural and synthetic form, the latter called epit(h)alon [174,175,176]. Besides lengthening the healthy life span, epithalamin could also reduce carcinogenesis, improve cardiovascular function, and protect retinal and brain function [174,176,177,178,179,180,181,182,183,184,185,186,187]. Epithalamin heightened peroxide chemiluminescence found in the blood of 30-month-old rodent models and humans [173]. Interestingly, epithalamin appeared to induce melatonin production [188,189,190]. Underscoring evolutionary conservation and strongly implying melatonin, the administration of pineal gland extract was found to extend life from humans to *Drosophila* [177,191,192,193,194,195].

Melatonin administration increased the life span of rodents [191,196,197,198], with an 18% extension reported for mice [171,172]. Melatonin was also found to increase the life span of several invertebrates, including *Drosophila* [196,199,200]. One study examining the protective effects of melatonin from ionizing radiation in rats observed that melatonin inhibited guanine base oxidation in DNA [201]. In *C. elegans*, it was found that genes related to mitochondrial function greatly affected life span [202]. RNAi inactivation pertinent to mitochondrial function, surprisingly, extended the life span of the average *C. elegans* [202].

#### Oxidative Damage

Oxidative damage is a major contributor to biological aging. The free radical theory of aging states that free radicals induce changes in cellular metabolism, which, eventually, leads to functional decline and organismal death through gradual loss in cellular function and reduced resistance against physiological stress [200,203]. ROS include oxygen-derived molecules and chemical species with one unpaired electron called free radicals [204]. ROS have been linked to oxidative damage of fatty acids, DNA, and proteins, as well as to the production of hydroxyl and peroxyl radicals, hydrogen peroxide, and superoxide radical anions [205]. ROS can also target mitochondria [206], which would induce mitochondrial stress and, eventually, reduce the life span [207]. The cellular capacity to counter oxidative stress is limited and may be overwhelmed when ROS form at an excessive rate [163,205] (Figure 2). Evidence of oxidative stress has been found in several age-related pathologies, including cancer, cardiovascular, inflammatory, and neurodegenerative diseases such as PD and AD [205,208]. Mitochondria are responsible for more than 90% of the oxygen consumption and, thus, produce the greatest amount of ROS [209]. Indeed, ROS can be formed through exogenous and endogenous means. Endogenous promoters of ROS formation include free radical semiquinone anion species (Q^−^) formed in mitochondria, and cytosolic intracellular enzymes, while extrinsic sources comprise environmental agents (e.g., ultraviolet light, ionizing radiation), including non-DNA reactive carcinogens and chemicals in pollutants such as methyl viologen, also known as herbicide paraquat [205]. Excess oxygen-containing compounds bring about tissue damage, chronic inflammatory processes, and disturb cell function [210]. Antioxidants and free radical scavengers, instead, inhibit the effects of free radicals because of their ability to quench oxidative stress.

Known for being a highly effective antioxidant and free radical scavenger [211], melatonin has been studied and used for anti-aging benefits and longevity extension to prevent oxidative and mitochondrial injury and maintain mitochondrial bioenergetics. Melatonin counters oxidative stress by working alongside other radical scavengers to donate electrons to unstable radicals [212,213,214]. It stimulates antioxidative glutathione peroxidase and glutathione reductase enzymes, as well as upregulates glutathione synthesis (reviewed in [163,215,216]). Melatonin can quench hydrogen peroxide, NO, peroxynitrite anion, superoxide anion, peroxyl, and the more damaging hydroxyl radicals, acting both in lipophilic and hydrophilic manners [215,217] (reviewed in [150,216]). In vitro, melatonin effectively reduced the peroxidation of ox-brain phospholipids [218]. Importantly, the breakdown metabolites formed during the quenching process, such as cyclic 3-hydroxymelatonin, N-acetyl-N-formyl-5-methoxykynuramine, and N-acetyl-5-methoxykynuramine, also donate electrons [219,220,221,222,223], creating an “antioxidant cascade” (reviewed in [163]), where free radicals are progressively eliminated. Resistance to oxidative stress is one of the longevity extending factors [200].

Aging decreases activity of the mitochondrial respiratory chain and slows down ATP production [224]. Steady melatonin administration in the drinking water of one-month-old mice until ten months of age appeared to effectively counteract the age-related decline of lung functionality and increase ATP production, indicating that melatonin administration maintained fully functioning lung mitochondria during aging [225].

### 3.2. Drosophila in Longevity Studies

*Drosophila melanogaster* is an ideal model species to specifically study longevity because of its relatively short life span, and conservation of ~75% of the genes and pathways involved in human disease and longevity [226,227]. With a rigorous comparative perspective, core biological mechanisms can be deciphered in the fly and the resulting knowledge may be used to inform complex vertebrate modeling. Fruit fly physiology can be directly (and carefully) compared to that of humans. Relevant for aging studies, *Drosophila* presents two sexes, sexual dimorphism enabling the examination of sex-specific differences, and highly differentiated tissues [228]. Economic culturing in the laboratory, without the need of expensive containment facilities, and the wealth of accessible genetic resources (e.g., public strain repositories, construct collections available to the research community at minimal cost) make *Drosophila* particularly amenable to studying the genetics of aging. Moreover, its short life span eases the study of potential life-extending drugs. *D. melanogaster*, as well as other invertebrate and unicellular model organisms, have first indicated that caloric restriction positively impacts longevity [229]. Adult *Drosophila* that were administered a mechanistic target of rapamycin (mTOR) inhibitor rapamycin through their food source, displayed the life span extension patterns observed in mTOR mutants [230]. Such effect is also visible in Figure 8 of Gamberi et al., 2017 [231]). *Drosophila* research confirmed the involvement of the nutrient-sensing insulin/insulin-like growth factor signaling (IIS) pathway in regulating aging. The mutational inactivation of three of the seven insulin-like peptides encoded in the fly genome (*dilp2–3,5*) yielded a dramatic 30–50% life span increase [232]. Another study provided evidence that *circ* RNA encoded by *sulfateless* (*circSfl*) is downregulated over time, contributing, significantly, to aging; however, *circ* RNA upregulation only increased the average life span of *Drosophila* by about 15% [233]. Underscoring mechanistic conservation, dietary restriction positively affected longevity in *Drosophila* [234]. However, how calorie restriction and altered macronutrient intake balance result in increased longevity, have not yet been identified [235].

## 4. Melatonin and Neurological Disease

Linked to melatonin function, many neurodegenerative diseases such as Alzheimer’s disease (AD), Huntington’s disease (HD), and Parkinson’s disease (PD) are associated with the disruption of the circadian clock and function [236] (reviewed in [237]). Moreover, oxidative damage is widespread among neurological diseases [238,239,240]. As well, defective mitochondrial function leading to oxidative imbalance is thought to lead to neurological disease pathology directly or indirectly (reviewed in [241]). It has recently been shown that the melatonin synthesized by the mitochondrial matrix can activate MT1 signaling which inhibits cytochrome *c* release and caspase activation, thereby halting neurodegeneration [19]. Thus, these pathologies may offer opportunities of intervention through melatonin or the modulation of melatonin-responsive pathways.

### 4.1. Alzheimer’s Disease

The hallmark of AD consists of the accumulation of extracellular senile plaques, composed of amyloid β peptides, and intracellular neurofibrillary tangles, composed of aggregated neuronal cytoskeletal tau protein [241,242,243]. AD is characterized by the disruption of cognitive functions and progressive memory loss [243] (reviewed in [244]). As well, mitochondrial abnormalities have been shown to play an important role in disease pathogenesis, often leading to the inhibition of electron transport in the brain, including reduced activity of cytochrome *c* oxidase [245] (reviewed in [241]). In AD, inhibited electron transport from the mitochondria is thought to yield oxidative imbalance favoring accumulated mitochondrial oxidants [241]. Melatonin has been shown to play a role in AD because it not only protects against oxidative stress, but also against amyloid β-peptides accumulation, which are typical of AD pathogenesis [243,246,247]. Suggesting a weaker melatonin signal transduction, patients suffering from AD also have reduced melatonin and MT1/MT2 receptor levels; more precisely, in AD patients, the immunoreactivity of both MT1 and MT2 receptors appeared distinctly decreased within the pineal gland cell somata and cellular processes [248].

### 4.2. Huntington’s Disease

Caused by mutations of the *Huntingtin* (*Htt*) gene, HD leads to motor impairment such as involuntary movements, cognitive impairment such as dementia, and psychiatric symptoms among which anxiety and depression are common (reviewed in [249]). From murine models, it has been found that Htt is necessary for mitochondrial metabolism and bioenergetics [250]. Just as AD, HD has also been associated with mitochondrial abnormalities such as a defective electron transport chain and Ca^2+^ uptake [251,252]. HD has also been associated with a reduction in nightly melatonin levels, accompanied by sleep and circadian function disturbances [253]. Diminished oscillations of core clock genes such as *period* and *timeless* have also been observed [236]. In a rat model of HD, melatonin treatment has been proposed to delay the onset of disease symptoms due to its antioxidant properties [254].

### 4.3. Parkinson’s Disease

PD is frequently caused by mutations in the *leucine-rich repeat kinase 2* (*LRRK2*) gene (reviewed in [255,256]) and it leads to severe motor symptoms such as tremors or slow movements and sleep disturbances [257]. Sleep disturbances are found in 60–98% of LRRK2-associated PD patients [258], suggesting the need for therapeutics, potentially targeting the genes and/or symptoms associated with sleep disorders. Melatonin supplements have been widely used to induce sleep and it is hypothesized that treatment in PD patients could normalize melatonin levels, inducing regular sleep patterns [257]. As well, PD patients were found to be defective in the mitochondrial Complex I, leading to abnormal electron transport, neuronal depolarization, impaired mitochondrial Ca^2+^ uptake, and oxidative imbalance [241,259,260,261,262]. PD pathogenesis also includes the impaired removal of defective mitochondria [241,263] and has been classified as a “mitochondrial disease” (reviewed in [264,265]).

## 5. Melatonin Life Extension in *Drosophila* Models of Aging and Disease

Both melatonin and arylalkylamine N-acetyltransferase, a key catalyst for melatonin synthesis, are conserved in flies [200,266]. Moreover, *Drosophila* extracts can synthesize melatonin from its natural building blocks tryptophan, tryptamine, and serotonin [266]. Melatonin fed to the *D. melanogaster* Oregon wild strain at a daily concentration of 100 μg/mL within the culture medium increased the maximum life span by 33.2%, compared to vehicle-fed flies [200]. Another study found that exposure of the *Drosophila* HEM strain to melatonin and pineal peptide epithalamin [173], increased the mean life span by about 17% [196]. Epithalamin administration increased antioxidant activity by 36.6% and SOD by 19.7% [173]. Suggesting mechanistic conservation, epithalamin was found to lengthen the *Drosophila* lifespan and reduce oxidative stress [267,268,269]. In flies, epithalamin appeared to promote transcription by favoring DNA strand separation at promoter sites while stimulating euchromatin formation, which is instead progressively lost during aging [270].

Melatonin dispensed to the *Drosophila* Canton-S wild strain at the early stages of development, and a concentration of 0.08% per unit mass in a culture medium, increased male life span by 15% [199]. Melatonin administration (0.43 mM) increased both life span and malondialdehyde levels [271]. Malondialdehyde reduces aging-related free radical damage, and its levels are used as a clinical indicator of antioxidant potential in patients [272]. As that of other insects, the *Drosophila* genome does not contain clear MT1 and MT2 homologs, although it encodes several orphan GPCRs and the downstream effectors of signal transduction. Worm and fly genomes include quinone reductases of distinct origin from NQO2 [273]. The *Drosophila* ROR homolog, *ultraspiracle*, has been studied for its endocrine function related to the hormone ecdysone [274,275], but it is known to be important for retinoid metabolism in eye development and tissue regeneration [276,277] both having functional conservation. Interestingly, retinoids are crucial for retinal development and appear linked to melatonin function [278]. However, functional relatedness and melatonin binding remain to be determined. Due to the complicated melatonin functional overlap in mammalians, *Drosophila* appears an ideal system in which to distinguish evolutionary recent GPCR receptor-mediated functions from ancient melatonin functional pathways.

### 5.1. Melatonin Treatments in Drosophila Models of Neurological Disease

With a nervous system similar to humans, yet streamlined [279,280], *Drosophila* models have recently been key to understanding HD pathogenesis and aspects of other neurodegenerative diseases [226,227]. The *Drosophila* circadian clock system contains fewer circadian pacemaker neurons than humans, which enabled functional studies and, likely, the most precise definition of the molecular, genetic, physiological, and behavioral aspects of any circadian clock system [281]. In the fly, the setup of the circadian rhythm is regulated largely at the transcriptional level by the daily expression cycle of several genes, including *period* (*per*) [282,283,284] and *timeless* (*tim*) [285], as well as the *pigment dispersing factor* (*pdf*) found in circadian pacemaker neurons [286] (reviewed in [287,288]). While the complex mammalian circadian rhythm relies on multiple clocks (reviewed in [289]), the basic organization of the oscillators is conserved between flies and mammals and several key proteins are also conserved. Therefore, *Drosophila* is considered a valid model in which to study neurodegenerative disease (reviewed in [290]). Importantly, the high conservation of oxidative response pathways makes *Drosophila* a useful model to further explore the role of oxidative stress in pathologies, including neurodegenerative and renal diseases [291,292,293,294] (Table 2).

#### 5.1.1. Huntington’s Disease

Very recently, transgenic HD flies were generated by expressing mHtt in pan neuronal and pdf-specific neurons [236]. The HD fly model displays the progressive loss of motor function and reduced oscillations of core clock genes *per* and *tim* [236]. The *Drosophila* HD model was used to investigate the effects of melatonin and curcumin, a compound found in turmeric plant roots, on eclosion and the characteristic progressive loss of locomotion [236]. Curcumin exhibits several therapeutic properties, e.g., antioxidant and anti-inflammatory, and, notably, it has been suggested to be neuroprotective for PD and AD [309]. This study found that melatonin significantly increased both percent eclosion and motor function measured as the climbing ability of the HD flies, compared to control flies of the same age [236]. Curcumin (10 μM) also improved the climbing ability of the HD flies. Neither melatonin nor curcumin had adverse effects on control flies [236], suggesting they may be non-toxic and suitable to long-term therapeutical administration. Moreover, similar to the aging process which features the progressive loss of circadian functions, HD flies aged 1 through 13 days normally displayed a lower amplitude of *per* and *tim* mRNA oscillations compared to the wild-type controls [236,310,311]. However, both melatonin and curcumin rescued the daily (24 h) *per* and *tim* mRNA oscillations to normal levels in HD flies [236]. Khyati et al. speculated that melatonin and curcumin supplementation to HD flies may prevent neurodegeneration by inhibiting oxidation and blocking Htt protein aggregation, respectively [236]. Consistently, in a mouse model of HD, Htt protein accumulation and aggregation, as well as transcriptional deficits, were present by six months of age and were both improved by dietary curcumin [312]. Oxidative damage is widespread in neurodegenerative disease [238,239,240] and the antioxidant properties of melatonin make it a promising therapeutic candidate. However, melatonin’s effectiveness appears to be due to more than its antioxidant function. Indeed, while oxidative damage appears important for HD fly pathology, supplementation with antioxidant SOD and dietary antioxidants α-tocopherol and coenzyme Q10 in HD flies was not enough to rescue the lethal HD phenotype [296]. This observation is reminiscent of the Martin and colleagues finding that in rat mitochondria, glutathione levels were responding specifically to melatonin and not to other antioxidants, i.e., ascorbic acid and α-tocopherol [147]. These observations in different models make it tempting to speculate that melatonin may have unique critical properties. A clue about melatonin’s mechanism of action in HD may come from the observation that the skin fibroblasts of HD patients displayed significantly reduced activity of antioxidant catalase [303]; therefore, melatonin may potentially be used to increase catalase activity in HD. Antioxidant enzymes influenced by melatonin are well conserved in *Drosophila* and can be further studied to gain mechanistic detail (Table 2). As well, the amelioration of HD pathology is likely to involve an improved regulation of core melatonin-responsive clock-gene pathways, that can also be rapidly characterized in *Drosophila* [17,236]. Overall, melatonin and curcumin both showed promise in ameliorating HD symptoms, and further studies are required to understand their precise action following circadian clock disturbances and the potential for synergistic or additive effects.

#### 5.1.2. Parkinson’s Disease

Recently, transgenic flies were generated by selectively expressing human (h) LRRK2 in mushroom bodies, where sleep is regulated in *Drosophila* [257]. “Humanized” LRRK2 transgenic flies recapitulate key properties of human PD such as motor impairment and the loss of dopaminergic neurons [257,313]. The hLRRK2-expressing flies displayed sleep fragmentation caused by elevated arousal at night, as well as the disturbance of presynaptic function demonstrated by the decrease in cellular excitability of Kenyon cells in mushroom bodies [257]. Melatonin treatment was found to ameliorate both conditions. The dark phase (night) mean sleep length was increased and the frequency of excitatory postsynaptic potentials (EPSPs) was restored to normal levels upon administering melatonin (4 mM) to the hLRRK2 transgenic flies [257]. The observed rescue was thought to reflect improved synaptic transmission due to a melatonin-dependent reduction in ROS, that would otherwise damage neurotransmitter release [257]. Similarly, SOD and catalase activity were found significantly decreased in PD patients as the disease progressed [295,314]. Melatonin treatment could also improve long-term memory deficits in hLRRK2 flies by regulating the presynaptic membrane Ca^2+^ activity of Kenyon cells, though exact mechanisms are still unknown [315]. More than 80% of PD patients experience cognitive decline often leading to long-term memory impairment [315,316]. Therefore, melatonin treatment could greatly improve their quality of life. Overall, melatonin seems to be a promising treatment for PD patients expressing LRRK2 mutations with severe sleep-related problems and cognitive decline.

#### 5.1.3. Alzheimer’s Disease

AD is identified by the accumulation of plaques composed of amyloid beta (Aβ) peptides [243]. One dominant form of Aβ peptides, Aβ42, is speculated to play an important role at the start of AD pathogenesis [243]. In AD, Aβ42 oligomers form interactions with mitochondrial proteins, leading to mitochondrial dysfunction and excessive ROS production (reviewed in [317]). Thus, antioxidants such as melatonin have previously been used to inhibit Aβ oligomerization in AD [318]. A recent study has used transgenic flies overexpressing human Aβ42 in the central nervous system to study the effects of melatonin. The treatment was found to have many benefits in the AD *Drosophila* model. Melatonin (0.43 mM) significantly improved climbing ability and increased the AD fly life span compared to untreated control flies [243]. As well, an immunoblot analysis showed that Aβ42 expression was reduced in flies exposed to melatonin [243]. Moreover, fluorescence assays displayed normalized ROS levels in the mitochondria of treated AD flies, compared to untreated ones that, instead, contained high levels of ROS [243]. Because of these promising results, melatonin seems to be a potential treatment for AD due to its antioxidant properties, although a deeper characterization of these effects is needed.

### 5.2. Melatonin Treatment in a Novel Drosophila Model of Polycystic Kidney Disease

Recently, we showed that melatonin reduced cysts in a first-in-kind *Drosophila* model of autosomal-dominant polycystic kidney disease (ADPKD) [319], which raises the novel and intriguing possibility that melatonin may be beneficial in PKD treatment. ADPKD is a genetic disease caused by mutations in genes *PKD1* (80% of all cases) or *PKD2* (15% of all cases) [320]. ADPKD is characterized by the progressive formation of fluid-filled cysts along the length of the renal tubules (nephrons). Cystic growth disrupts normally regulated proliferation and apoptosis of the epithelial cells forming the renal tubule and it displays some neoplastic characteristics (reviewed in [321]). The first-in-kind *Drosophila* model of PKD harbors a mutation in the *Bicaudal C* (*BicC*) gene [231]. Conserved evolutionarily from flies to humans, the human *BicC* ortholog is called *BICC1* and the murine one *Bicc1*. Both *BICC1* and *Bicc1* appear to be genetically downstream of key PKD gene *PKD1* [231]. The mutation of any vertebrate *BicC* genes was sufficient to induce renal cysts (reviewed in [322]), implying that *BicC* dysregulation may contribute to renal cystic pathogenesis in conditions of *PKD1* loss-of-function.

The *Drosophila* PKD model was developed by crossing flies containing a *BicC* deletion (*Df(2 L)RA5/CyO* or *Δ*) with flies containing a *BicC* hypomorphic mutation (*BicC^YC33^/CyO*), generating *BicC^Δ/YC33^* mutant flies that recapitulate several phenotypic and molecular aspects of human PKD, such as the formation of fluid filled cysts along the renal (Malpighian) tubules, as well as increased activity of the mTOR kinase and *myc* upregulation, which both control cell proliferation and apoptosis [231,323]. *BicC* mutants also show conserved pharmacological response to Smac mimics [324,325] and rapamycin [231]. Melatonin (150 μM) was administered at night to the *BicC^Δ/YC33^* mutant flies aged 0–2 days and Malpighian tubules were dissected after 18 days of treatment [319]. Melatonin was found to significantly reduce cysts by over 30% along the entire tubule length of *BicC^Δ/YC33^* flies, compared to vehicle-treated flies [319]. *Drosophila* features one longer anterior and one shorter posterior Malpighian tubule pair, each with different transcriptomes and functions [326]. As well, each tubule pair contains three functionally distinct regions dubbed proximal, intermediate, and terminal (reviewed in [327]). Melatonin-promoted cyst reduction affected such regions differentially [319]. Compared to vehicle-treated controls, the proximal region of melatonin-treated flies was rescued most effectively with a 59% cyst reduction [319]. The intermediate and terminal regions showed 37 and 31% reduction, respectively [319]. These results highlight the functional differences occurring along the anterior and posterior tubule regions [319,328] (reviewed in [327]) and raise the intriguing possibility that such differences may be conserved to humans. Although not extensively studied, melatonin has been found to support normal kidney function in mammals. Mesenchymal stem cells pre-treated with melatonin and transplanted into the kidneys of a rat model of chronic kidney disease (CKD) displayed less disturbance of the basement membrane and improved renal tubule histology, while reducing overall fibrosis, a common complication [329,330]. As well, the preconditioning of the mesenchymal stem cells with melatonin reduced transforming growth factor (TGF)-β, tumor necrosis factor (TNF)-α, and α-smooth muscle actin expression, while increasing E-cadherin expression, indicating the amelioration of cell-to-cell adhesion in the tubules [329]. It was suggested that TNF-α reduction may be due to melatonin antioxidant activity, which decreases ROS production and inflammation [329,331], but additional investigation is needed to fully characterize the molecular mechanism.

While the potential mechanism of action for melatonin in renal cyst reduction is not known, one important feature of ADPKD is oxidative stress [297,299,332], which could be attenuated by the antioxidant properties of melatonin. Oxidative stress markers such as 8-isoprostane, asymmetric dimethylarginine and prostaglandin PGF_2a_ have been shown to be elevated in ADPKD patients with a preserved estimated glomerular filtration rate (eGFR), as compared to normal individuals [332] (reviewed in [333]). Reduced levels of SOD, as well as glutathione peroxidase were also found in ADPKD patients [297,302]. Consistently, murine models of ADPKD featured decreased antioxidant enzymes glutathione peroxidase, catalase, glutathione S-transferase, and SOD, while displaying aggregates of lipid peroxidation byproducts in plasma and kidneys [299]. Moreover, murine models of ADPKD suggest that mitochondrial dysfunction may also play a role in inducing oxidative stress leading to cyst formation [334]. Mitochondrial function seems indirectly regulated by the PC1–PC2 complex, which promotes mitochondrial Ca^2+^ uptake and regulates oxidative phosphorylation [335]. Suggesting that renal cystic diseases may share a signature of oxidative stress, rat models for the autosomal recessive (AR) form of PKD also feature increased oxidative stress [336]; however, more markers need to be studied for this condition and to clarify mitochondrial involvement. Because the evolutionary ancient oxidative response pathways are conserved in *Drosophila* [291,292,293,294] (Table 2), *Drosophila* seems ideal to model how melatonin affects PKD-type cysts. Melatonin is also known to regulate several pathways implicated in neoplastic growth (e.g., mTOR (reviewed in [337]), MAPK [338,339], JAK/STAT3 [339,340,341], ERK [337,342], and TGF-β/Smad [343]). Several of these pathways are also altered in ADPKD tissue and related neoplastic renal cell carcinoma (reviewed in [321,344]). Thus, the observed improvements in the melatonin treated cystic *BicC* flies may result from the simultaneous correction of several cellular defects. Another possible contributing mechanism, ADPKD, similar to several other diseases, including cancer, causes a profound metabolic reprogramming of the renal cystic cells and defective glucose metabolism [345] such as or similar to the Warburg effect [346,347]. In these conditions, pyruvate, that is normally transported to the mitochondria, remains in the cytoplasm, and is fermented to lactate in hypoxic conditions. Melatonin appears to counteract the Warburg effect by inhibiting the hypoxia inducible factor (HIF) 1α, which increases pyruvate uptake by the mitochondria and its conversion to acetyl-coenzyme A [348]. As mentioned above, acetyl-coenzyme A is also a cofactor for melatonin synthesis, which consolidates the pathway. These melatonin-mediated effects have been found to rescue cell metabolism in oncology [349] and may also contribute to ameliorating the Warburg-like effects in the renal cyst and ADPKD. Considering that all these pathways are well-conserved in *Drosophila* (reviewed in [321,327,350]), and corresponding gene knockout and knockdown flies exist, it should be possible to decipher the molecular bases of melatonin efficacy as a cyst-reducing drug candidate. Overall, our *Drosophila* studies gave insight into a potential novel treatment for ADPKD, that being non-toxic, could likely be administered indefinitely to alleviate symptoms of chronic ADPKD. In oncology, melatonin also exhibited promise as a combination drug that potentiates chemotherapy and, simultaneously, shields normal tissue from its damaging effects [351,352,353,354,355,356,357,358,359,360,361]. Possibly, the melatonin ability to alleviate the side effects of other drugs may be tested in combination with tolvaptan, the only approved ADPKD drug that has displayed signs of potential hepatotoxicity [362,363,364]. While intriguing, further studies will be needed to fully determine the efficacy and applicability of melatonin treatment to ADPKD therapy.

## 6. Discussion

Melatonin is a universally conserved molecule and major regulator for (virtually) all biological organisms. It displays evolutionary ancient functions as an antioxidant and transcriptional regulator that protects both unicellular and multicellular organisms from endogenous and exogenous stress and oxidative injury. In the latter organisms, melatonin has acquired additional functions to synchronize gene expression with daily and seasonal variations of the light/dark cycle. Overall, melatonin affects key aspects of metabolism, longevity, and biological adaptation. Mostly studied in mammals, the evolutionary recent functions are mediated through multifunctional MT1 and MT2 receptors. Belonging to the GPCR family, MT1 and MT2 can homo- and hetero-dimerize with each other and heteromerize with other receptors, displaying remarkable functional diversity. Through such key roles, melatonin appears to be a master regulator fine-tuned to species-specific biology and conceivably holds immense therapeutic potential. It is, therefore, unsurprising to find disrupted melatonin-related pathways in several diseases. Circadian disruption is known to occur in neurodegenerative diseases such as AD, PD, and HD; however, altered light/dark cycles have been observed in several other conditions that are seemingly unrelated to the CNS, including ADPKD. Increased oxidative stress, and mitochondrial dysfunction, conditions underlying several diseases, could be improved through melatonin-induced renormalizing functions. Being non-toxic, melatonin makes it ideal for the protracted treatment needed in chronic disease. Speculatively, the melatonin capacity to affect and rebalance a multitude of pathways also makes it an ideal drug, especially in conditions causing metabolic reprogramming such as ADPKD and cancer. However, the incomplete knowledge of the myriad of melatonin roles and dosage effects still limits precise manipulation in diseased states. The potential for melatonin treatment is widely recognized in neurological disease and oncology, there is new promise for ADPKD and there appears to be an untapped capacity also in renal cell carcinoma, a malignancy sharing pathological aspects with ADPKD (reviewed in [321,344]). Considering that the complexity and redundancy of the mammalian melatonin pathways substantially complicate mechanistic studies, model organisms may be deployed to probe specific questions and mechanisms. Evolutionary conservation and a vast arsenal of genetic tools make *Drosophila* a key organism in which to distinguish core conserved ancient roles from those evolutionarily more recent. Due to the lack of bona fide MT1 and MT2 receptors, the powerful *Drosophila* genetics could potentially be used to generate humanized flies expressing human MT1 or MT2 receptors alone and in combination to test genetic interactions and gene modifiers in the whole organism and in specific tissues. Combined with up-and-coming fly pharmacology (reviewed in [227,350]) and toxicology [365,366], fly research promises to yield new biological knowledge with translational significance.

## Figures and Tables

**Table 1:** Melatonin receptor functions.

Receptor	Target	Function	Reference(s)
MT1	Pituitary	Decreases functions of luteinizing hormone (LH), follicle-stimulating hormone (FSH), and prolactin (PRL)	[65,66,67,68]
MT1	Testes	Decreases testosterone	[69]
MT1	Adrenal cortex	Reduces cortisol secretion	[70,71]
MT1	Metabolism	Limits insulin secretion and increases leptin production	[72,73]
MT1/MT2	Vasculature system	MT1 inhibits vasoconstriction; MT2 inhibits vasodilation	[74,75,76,77,78]
MT1/MT2	Cancer cells	MT1 reduces proliferation of cancer cells; MT2 reduces the proliferation of JAr cells	[79,80]
MT1/MT2/MT3	Immune system	MT1 counteracts prostaglandin E (PGE) inhibition of interleukin (IL-2) production; MT2 increases B splenocyte proliferation, anti-keyhole limpet hemocyanin (KLH) IgG levels and decreases leukocyte rolling; MT3 is responsible for leukocyte adhesion	[81,82,83,84,85]
MT1	Mammalian pituitary and SCN	Inhibition of cAMP accumulation by pertussis toxin (PTX)-sensitive G proteins	[26,41,48,86,87]
MT1		Melatonin binding activates Gi proteins which inhibits adneylyl cyclase activity and decreases cAMP	[38,46]
MT1	Inner Rectifier Potassium (Kir) channels, phosphorylation of mitogen-activated protein kinase (MAPK), and extracellular signal-regulated kinase 1 and 2	Increases potassium conductance	[38]
MT2	SCN	Inhibits forskolin-stimulated cAMP production in addition to cGMP formation and activation of PKC	[38]
MT1		Activation of multiple types of G proteins	[34]
MT2		Inhibition of adenylyl cyclase and guanylyl cyclase, as well as phosphoinositide production	[48,88]

**Table 2:** Antioxidant enzymes influenced by melatonin *: Implications in *Drosophila* disease models.

Enzymes	*Drosophila* Homolog	HD	PD	ADPKD	References
Superoxide dismutase (SOD)	Sod	No effect on neurodegeneration	Reduced activity as PD progresses	Reduced levels	[295,296,297]
Glutathione peroxidase (GPx)	PHGPx	Neuroprotective in different models of HD	Reduced activity possibly leading to dopamine neuron loss	Reduced activity	[298,299,300,301]
Catalase (CAT)	Cat	Reduced activity	Low activity possibly due to CAT inhibition by α-synuclein	Reduced activity	[302,303,304]
Glutathione reductase (GR)	-	Reduced activity causing redox imbalance	Increased levels suggesting attempt to maintain glutathione levels	Activity inhibited by acid pH in proximal tubules	[305,306,307,308]

* [163].

## Data Availability

Not applicable.
